# Standardization and professionalization of simulated and standardized patient-based education in Qatar: A call for action

**DOI:** 10.5339/qmj.2025.33

**Published:** 2025-06-09

**Authors:** Nandini Alinier, Guillaume Alinier

**Affiliations:** 1 Freelance Educator, Doha, Qatar; 2 Hamad Medical Corporation Ambulance Service, Doha, Qatar; 3 Weill Cornell Medicine – Qatar, Doha, Qatar; 4 School of Health, Medicine, and Life Sciences, University of Hertfordshire, Hatfield, UK; 5 Faculty of Health and Life Sciences, Northumbria University, Newcastle Upon Tyne, UK *Email: dinipem@yahoo.com

**Keywords:** Simulation, education, simulated patient, standardized patient, Qatar Simulation Consortium, symposium, Simulated participant

## Abstract

Simulation, in its various forms, is widely used in all stages of education, training, and assessment of healthcare students and professionals. There is, however, a lot of variation in practice regarding the way in which institutions implement simulation, and in particular how educators work with simulated participants (SPs). This short article makes a case for bringing together all of Qatar’s healthcare educators and SPs at the 2025 Qatar Simulation Symposium in order to enhance simulation-based educational practices and raise awareness of standards of best practice. It could play a significant role in improving learners’ experiences and eventually positively impact on patient care.

The adoption of simulation in healthcare education has not occurred uniformly worldwide for various reasons, including cultural and financial factors, as well as a lack of awareness or understanding of the various possible simulation modalities among educators. We aim to address this issue in Qatar, particularly regarding the use of lay or specifically trained individuals portraying the role of patients during various types of educational interventions or formal assessment sessions, at the upcoming annual simulation symposium. Although it is no longer a true innovation as a training modality in most countries, its relatively recent adoption in Qatar by several institutions could be enhanced.

Although the FIFA World Cup Qatar 2022™ fever is now long past, the country is still seeing some developments in several domains, notably in the education and healthcare sectors, with the recent opening of new hospitals and the creation of new health related educational programs to meet the demand for a larger locally trained healthcare workforce. One of the priority areas of Qatar’s National Health Strategy 2024–2030 is Service Delivery, which notably focuses on healthcare professionals and patient experience, both of which are directly related to education.^1^ Healthcare students and clinicians need to acquire and maintain a wide range of technical and non-technical skills, which necessitates the use of various educational approaches and tools.^2^

Clinical skills centers and simulation training facilities generally house a wide range of simulators and simulation-related technology ranging from part-task trainers (synthetic body parts and limbs), virtual reality or box trainers for procedural training, to full-body computer-controlled patient simulators (interactive manikins), which are all useful for learners to develop and refine their skills individually or sometimes as part of a multiprofessional team.^3^ Another key component is “actors”, commonly referred to as “simulated patients”, “standardized patients”, or even “simulated participants” (SPs), to encompass other possible roles during a simulation-based activity.^4^, They are individuals trained and paid to act as patients during educational encounters with learners and can give a full medical history based on a script. They may be used to teach various aspects of physical assessment and to practice non-invasive procedures and communication skills. When combined with another simulation adjunct for learners to practice invasive procedures, this approach is referred to as a hybrid simulation modality.^7^ Simulated patients are typically considered to have a less prescriptive role, such as during formative encounters (performed for educational purposes) and physical assessment practical sessions, whereas standardized patients or participants are expected to act according to a precise script to provide a uniform learning experience for all learners. This is particularly important for summative assessments, which are conducted for evaluation purposes or high-stakes assessments.

Although the use of SPs is often attributed to the work of Dr Barrows from the 1960s,^9^ the use of lay persons acting as patients in an educational context has been implemented earlier. One such example is the creation in the UK of Rescue Schools during the Second World War, in 1940, and the associated “Casualties Union” in 1942 which relied on volunteers applying moulage (make-up) to realistically simulate all sorts of wartime traumatic injuries and being positioned in precarious environments simulating battlefield conditions.^10^ The work was pioneering and provided a means for rescuers to learn to manage various severe injuries, to prepare themselves to deal with the associated psychological trauma of treating such types of casualties, and eventually save lives. Over 80 years ago, it was already recognized that these simulated casualties required training. The Casualties Union continues to actively serve the community by providing trained simulated patients for disaster preparedness exercises and supporting the training of healthcare professionals and rescue instructors.^11^

Despite the creation of the Qatar Simulation Consortium (QSC) in 2012, it remained a relatively informal network of simulation enthusiasts. The founding members began by hosting simulation facilitator workshops, which subsequently expanded from 2018 to become an annual symposium, enabling more participants to attend. Qatar’s healthcare simulation education community is still relatively nascent, with some institutions being more established and advanced than others in their implementation of various simulation modalities. Although simulation is not a mandatory training component in all academic healthcare educational programs, SPs are now used in all local courses during initial undergraduate training as well as in many continuing professional development courses, with considerable variations. In Qatar, the vast majority of SPs are hourly-paid freelance individuals rather than permanent employees of the institutions for which they work. They are regularly called upon for patient physical examination and communication exercises during formative and summative Objective Structured Clinical Examination sessions, and this was the case even during the COVID-19 pandemic.^12^ Expectations of SPs differ significantly from institution to institution, as do the methods by which they are recruited and assigned to learners’ training or assessment sessions, their training, and the level of engagement they have with learners. A significant amount of practical knowledge and experience can be shared by simulation educators regarding their use of SPs across a wide range of curricula. Similarly, considering the perspectives of the learners and the SPs themselves in the context of Qatar could be enriching and transform how most institutions work with SPs. For these reasons, we suggest that the next Qatar Simulation Symposium focuses on the SP modality to raise educators’ awareness about how they can help students and healthcare professionals practice delivering patient-centered care. It could also be an opportunity to bring together all current SPs in Qatar for their professional development by attending various workshops, as well as provide a forum for them to share their experiences and perspectives as SPs. We also encourage clinical educators in Qatar to engage with the broader simulation-based education community and the related annual conferences, especially those of the Association of Standardized Patient Educators (ASPE).^13^

In general, depending on the scenario or type of activity, SPs do not portray patients only. Instead, they may also portray other characters, such as family members or healthcare professionals, to ensure that very specific learning objectives related to particular situations can be addressed.^14^ To support educational institutions in their endeavors to enhance their simulation practice, including the use of SPs and their learners’ experience (both during teaching and assessment sessions), various resources are available. The International Nursing Association for Clinical Simulation and Learning, along with the Healthcare Simulation Standards of Best Practice^®^ and the ASPE Standards of Best Practice (SOBP) provide a good starting point.^15,16^ The five underlying values that inform this SOBP are: safety, quality, professionalism, accountability, and collaboration. The SOBP is also described by five domains of best practice: safe work environment, case development and SP training for role portrayal, feedback and completion of assessment instruments, program management, and professional development. They also recommend that activities and scenarios, especially those involving SPs, should be pilot-tested, which is particularly useful for SPs so they can assimilate their role. This also provides an opportunity for educators to check the quality of their role portrayals and standardize their performances.^17^ Simulation educators working with SPs would benefit from familiarizing themselves with these SOBP guidelines, as they facilitate the safety of participants and the delivery of effective simulation sessions. As a stepping stone towards achieving this goal across academic institutions that provide healthcare education in Qatar, we believe that focusing the theme of the next QSC symposium on the SP modality would promote a more coherent approach to its use across the various institutions that constitute Qatar’s Academic Health System.^18^ It could significantly contribute to the creation of a community of professional SPs with common values, standards, and ethics,^15^ which would in turn improve learners’ experience when interacting with them, contribute to the professional development of simulation educators in the country, and ultimately benefit patients.

The triad of people involved in the SP modality (Educator/Actor/Learner) creates many intricacies that are sometimes underestimated in achieving an optimal learning experience. Preparation in the broadest sense of the term, of physical and human resources, is key to all simulation-based activities. Looking outward and collectively reflecting on our current educational practices about the SP simulation modality could significantly enhance simulation-based practice in Qatar, notably by making it the central topic of the upcoming symposium and ensuring all stakeholders can attend and contribute.

## Conflicts of interest

Dr. Nandini Alinier serves as a simulated and standardized patient for multiple institutions in Qatar. Prof Guillaume Alinier is one of the Founding Members of the Qatar Simulation Consortium.
